# Coculture with Late, but Not Early, Human Endothelial Progenitor Cells Up Regulates IL-1****β**** Expression in THP-1 Monocytic Cells in a Paracrine Manner

**DOI:** 10.1155/2013/859643

**Published:** 2013-12-09

**Authors:** Qiuwang Zhang, Ivana Kandic, Jeffrey T. Barfield, Michael J. Kutryk

**Affiliations:** Division of Cardiology, Keenan Research Center, Li Ka Shing Knowledge Institute, St. Michael's Hospital, University of Toronto, Room 7084 Bond Wing, 30 Bond Street, Toronto, ON, Canada M5B 1W8

## Abstract

Endothelial progenitor cells (EPCs) have been used in clinical trials to treat ischemic heart disease. Monocyte infiltration plays an important role in inflammation, angiogenesis, and tissue repair during tissue ischemia. It is important to understand the interactions between EPCs and monocytes. In this study, a human EPC/THP-1 monocytic cell coculture system was used to examine EPC effect on IL-1**α**, IL-1**β**, and TNF-**α** expression in THP-1 cells. Late, but not early, EPCs upregulated IL-1**β** expression at both mRNA and protein levels. In contrast, neither early nor late EPCs affected IL-1**α** or TNF-**α** expression. Coculture with human umbilical vein endothelial cells did not alter IL-1**β** expression. It has been shown that activation of integrin **β**2 in human neutrophils augments IL-1**β** synthesis; however integrin **β**2 was not involved in IL-1**β** expression in THP-1 cells. Addition of late EPC conditioned medium to THP-1 cell culture led to a modest increase of IL-1**β** mRNA levels, indicating that late EPCs upregulate IL-1**β** expression partly through a paracrine pathway. IL-1**β**, an important inflammation mediator, has been shown to promote EPC function. Our data therefore suggest that late EPCs can exert self-enhancement effects by interacting with monocytes and that EPCs might modulate inflammatory reactions by regulating IL-1**β** expression in monocytes.

## 1. Introduction

Endothelial progenitor cells (EPCs), first described by Asahara et al. [1], represent a heterogeneous cell population derived from circulating CD34 positive or CD34 and KDR/VEGF receptor-2 (KDR/VEGFR2) double positive mononuclear cells. They have the ability to differentiate into endothelial lineage cells and have been shown to incorporate into newly formed vessels in animal models of ischemia. It has also been shown that CD133 positive cells demonstrated endothelial progenitor capacity [2, 3]. Extensive in vitro and in vivo studies have established that EPCs play an important role in vascular repair and regeneration [4–6], and several clinical trials have been completed that have assessed the efficacy of EPC therapy in patients suffering ischemic heart disease [7–10].

Currently, the most common protocol to obtain EPCs is through culture of peripheral blood derived mononuclear cells (PBMNCs) in endothelial cell (EC)-specific media [11]. This method has led to the identification of 2 distinct populations of EPCs, that is, early and late EPCs [12]. Early EPCs demonstrate a typical spindle-like morphology and are generated after culture of PBMNC for 4–7 days. They possess little proliferative ability but robustly produce angiogenic growth factors such as VEGF, hepatocyte growth factor (HGF), and IL-8. Cobblestone-shaped late EPCs emerge after culture of PBMNCs for 2–4 weeks and show much higher rates of proliferation. Both early and late EPCs have been shown to contribute to vascular repair and regeneration although their angiogenic properties are different. Early EPCs exhibit significant paracrine effects while late EPCs incorporate into vessels with minimal secretion of angiogenic factors [12, 13].

Monocyte infiltration plays an important role in inflammation, angiogenesis, and tissue repair and may influence the pathophysiologic processes involved in tissue ischemia. After accumulation in ischemic zones, monocytes are activated and produce cytokines to regulate disease process, which include tumor necrosis factor-alpha (TNF-*α*) and IL-1 (IL-1*α* and IL-1*β*). Tissue ischemia also leads to the mobilization of EPCs from bone marrow into circulation. The circulating EPCs then home to sites of ischemia to aid tissue repair through neovascularization [5, 14–17]. These EPCs may interact with the infiltrating monocytes in the ischemic zones. TNF-*α*, transforming growth factor-beta (TGF-*β*), and interleukins (ILs) are recognized as the major mediators of tissue ischemia [18, 19], among which TNF-*α* and IL-1 are considered to be the most important [19]. In light of continued interest in using EPCs to treat ischemic heart disease, it is important to understand interactions between monocytes and EPCs. Therefore, this study was conducted to examine the effects of EPCs on the monocytic expression of IL-1*α*, IL-1*β*, and TNF-*α*, using a human EPC/THP-1 monocytic cell coculture system.

## 2. Materials and Methods

### 2.1. Human EPC Isolation

Informed written consent for study participation was obtained from all volunteers. A total of 10 healthy individuals were recruited (5 males and 5 females with an average age of 38.9 ± 7.8 years). All protocols involving human samples were approved by the Research Ethics Board of St. Michael's Hospital, University of Toronto, in accordance with The Code of Ethics of the World Medical Association (Declaration of Helsinki). PBMNCs were isolated from healthy volunteers by Ficoll gradient centrifugation as described previously [20]. The PBMNCs were plated at a density of 0.75 × 10^6^ cells/cm^2^ in human fibronectin-coated flasks and cultured in endothelial growth medium supplemented with VEGF, basic fibroblast growth factor, and insulin-like growth factor-1 (EGM-2 medium, Lonza). After 3 days, nonadherent cells were removed and fresh culture medium was supplied. At day 7, cells were considered early EPCs. To obtain late EPCs, cells were maintained in EGM-2 medium with change of medium every other day until sporadic EPC colonies began to form. These colonies were then detached with 0.05% trypsin/1 mM EDTA (Life Technologies), pooled, and expanded. At day 28, cells were considered late EPCs.

### 2.2. Cell Culture

Human THP-1 monocytic cells (ATCC) were maintained in RPMI 1640 media with 10% FBS as recommended by the supplier. Human umbilical vein endothelial cells (HUVECs, Lonza) were maintained in the endothelial basal medium EBM-2 (Lonza) containing 10% FBS.

### 2.3. Characterization of EPCs

Early EPCs were characterized by uptake of fluorescent dye Dil-labeled acetylated low-density lipoprotein (Dil-Ac-LDL) and binding of FITC-labeled UEA-lectin (FITC-UEA-Lectin) as described previously [11, 21]. Late EPCs were examined for the expression of EC protein markers, that is, KDR/VEGFR2, Tie2, and eNOS, by western blotting. Western blotting analysis was performed as follows: 100 *μ*g of late EPC protein in 1X Laemmli buffer was boiled for 5 minutes, separated by SDS-PAGE, and electrically transferred onto nitrocellulose membrane. The membranes were blocked in TBS-T buffer (50 mM TrisHCl, 150 mM NaCl, pH 7.5, 0.1% Tween-20) containing 5% skimmed milk for 1 hour at room temperature followed by incubation for 2 hours with first antibodies diluted in block buffer. The first antibodies were used as follows: anti-human KDR/VEGFR2 polyclonal antibody (1 : 1000, Upstate); anti-human Tie2 polyclonal antibody (1 : 1000, Santa Cruz); and anti-human eNOS monoclonal antibody (1 : 2000, BD Biosciences). After incubation with the first antibody, the membranes were washed 3 times, each time 15 minutes with TBS-T buffer followed by incubation with HRP-conjugated anti-mouse or anti-rabbit secondary antibodies (Promega, both in dilution of 1 : 5000) for 1 hour. The membranes were washed as above with TBS-T buffer and the specific bands were visualized by an ECL Western Blot Detection Reagents Kit (Amersham Biosciences), scanned, and documented with Molecular Analysis Program (Bio-Rad Laboratories).

### 2.4. Coculture of THP-1 Monocytic Cells with Human EPCs

Early or late EPCs were detached with 0.05% trypsin/1 mM EDTA and seeded at 2 × 10^5^ cells/well into 6-well plates. 24-hour culture was carried out to allow EPCs to firmly adhere to the plate. After one wash of EPCs with PBS, THP-1 monocytic cells (4 × 10^5^ cells/well) were added. For all coculture and control culture experiments, RPMI 1640 medium with 10% FBS was used. Following culture for 24 hours, coculture conditioned medium (CM) with suspended THP-1 cells was transferred to a microtube followed by a centrifugation at 500 g for 5 minutes. The pelleted THP-1 cells were then used for total RNA extraction and the conditioned medium collected for detection of IL-1*β* protein by an ELISA Kit (R&D Systems). Culture of THP-1 cells alone was used as control. A similar coculture of HUVEC/THP-1 cells was performed to examine HUVEC effects on gene expression of IL-1*β*. To examine if integrin *β*2 in THP-1 cells has a role in late EPC regulated IL-1*β* expression, THP-1 cells were preincubated with an integrin *β*2-blocking antibody (clone TS1/18, 10 *μ*g/mL, BioLegend) for 1 hour, and then added to late EPCs for co-culture as described above. To study if late EPCs regulated IL-1*β* expression through a paracrine pathway, 1 mL of late EPC CM was added to THP-1 cell culture and early EPC CM was used as control.

### 2.5. Total RNA Extraction

Total RNA was extracted from THP-1 cells using a miRNeasy Mini Kit (Qiagen) according to the manufacturer's instructions. Briefly, the cell pellet was lyzed with 700 *μ*L QIAzol Lysis Reagent and the cell lysate mixed with 200 *μ*L of chloroform followed by centrifugation at 12000 rpm for 15 minutes. The aqueous phase containing RNA was transferred into a nuclease-free tube and mixed with 1.5 volumes of 100% ethanol. The sample was then passed through the RNeasy minispin column. After washes with RWT and RPE Buffers, RNA was eluted with 40 *μ*L of nuclease-free water and quantified with a spectrophotometer (BioRad Laboratories).

### 2.6. Quantitative Reverse Transcription-Polymerase Chain Reaction (qRT-PCR)

The first strand cDNA was generated by reverse transcription (RT) using an Omniscript RT Kit (Qiagen). The RT reaction, in a total volume of 20 *μ*L, contained the following components: 1 *μ*g of total RNA, 2 *μ*L of 5 mM dNTPs, 5 *μ*L of random primer (300 ng/mL), 2 *μ*L of 10x reaction buffer, 1 *μ*L of Reverse Transcriptase (4 units), 1 *μ*L of RNase inhibitor (1 unit), and nuclease-free ddH_2_O (used to adjust the final reaction volume). RT was accomplished by incubation at 37°C for 1 hour followed by enzyme inactivation at 65°C for 15 minutes. For real-time PCR, a total reaction volume of 20 *μ*L was achieved by mixing 2 *μ*L of RT product, 10 *μ*L of 2x SYBR Green PCR Master Mix, 200 nM of forward and reverse primer mix, and appropriate volume of nuclease-free ddH_2_O. Real-time PCR was performed on a 7900 HT Sequence Detection System (Applied Biosystem Inc.) in following stages: stage 1: 50°C for 2 minutes, stage 2: 95°C for 10 minutes, and stage 3: 95°C for 15 seconds followed by 62°C for 1 minute. Stage 3 was repeated for 40 cycles. A dissociation curve was set up for each reaction to examine the specificity of gene amplification. In addition, to eliminate the detection of genomic contamination, the pair of primers for each individual gene was designed to span an intron. Glyceraldehyde-3-phosphate dehydrogenase (GAPDH) was used as an internal control and the relative mRNA levels of IL-1*α*, IL-1*β*, and TNF-*α* normalized to GAPDH were quantified using the 2^−ΔΔCT^ method. Primer sequences were as follows:

 IL-1*α* (gene accession number: NM_000575.3),
 sense 5′-CCTCCATTGATCATCTGTCTCT-3′ and
 antisense 5′-CTCAACCGTCTCTTCTTCAGGA -3′;
 IL-1*β* (gene accession number: NM_000576),
 sense 5′-AACCTCTTCGAGGCACAAG-3′ and
 antisense 5′-GTTTAGGGCCATCAGCTTCA-3′;
 TNF-*α* (gene accession number: NM_000594),
 sense 5′-CCCAGGCAGTCAGATCATCTTC-3′ and
 antisense 5′-AGCTGCCCCTCAGCTTGA-3′;
 GAPDH (gene accession number: x02231),
 sense 5′-CTCTAAGGCTGTGGG CAAGGTCAT-3′ and
 antisense 5′-GAGATCCACCACCCTGTTGCTGTA-3′.

### 2.7. Enzyme Linked Immunosorbent Assay (ELISA)

ELISA was performed to measure IL-1*β* protein levels in the late EPC/THP-1 cell coculture conditioned medium. A high sensitivity human IL-1*β* ELISA Kit with a minimum detectable dose <0.1 pg/mL was obtained from R&D Systems. The ELISA procedure was accomplished following the manufacturers' instructions.

### 2.8. Statistical Analysis

Data were expressed as mean ± standard error of the mean (SEM). Statistical significance was determined using Student's *t*-test; *P* < 0.05 was considered statistically significant.

## 3. Results 

### 3.1. Isolation and Characterization of Human EPCs

**Figure 1:**
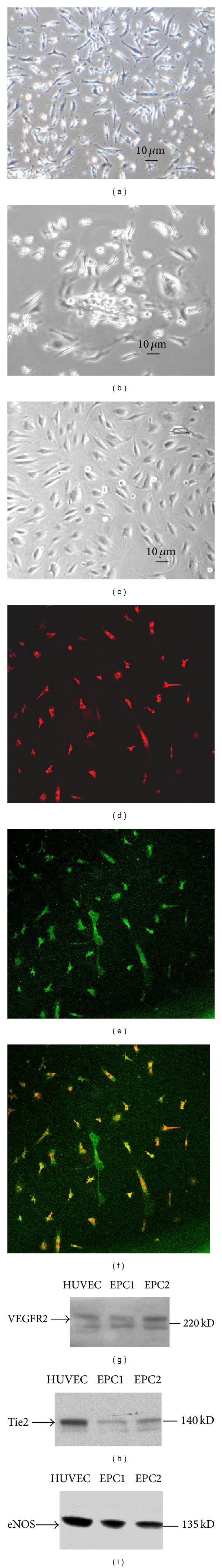
Culture and characterization of human EPCs. EPCs were generated by culture of PBMNCs in EC growth medium (EGM-2 medium). Spindle-shaped early EPCs appeared in culture at days 4–7 (a). At around week 2, sporadic cell colonies formed (b), which were expanded to generate cobblestone-shaped late EPCs (c). Early EPCs were characterized by cell uptake of Dil-Ac-LDL ((d) red) and stain of FITC-UEA-lectin (e) green). Over 80% early EPCs were Dil-Ac-LDL/FITC-UEA-lectin double positive ((f) orange). Late EPCs expressed a series of EC protein markers, that is, VEGFR2 (g), Tie2 (h), and eNOS (i), as determined by western blotting. Human umbilical vein endothelial cells (HUVEC) were used as control for 2 isolations (EPC1 and EPC2) of late EPCs.

Semiconfluent spindle-shaped early EPCs appeared after a 7-day culture of PBMNCs in the endothelial selective medium- EGM-2 (Figure 1(a)). At around week 2, sporadic cell colonies started to emerge (Figure 1(b)). These colonies were detached with trypsin/EDTA and replated for cell expansion. Cobblestone-shaped late EPCs proliferated rapidly, while spindle-shaped early EPCs died out almost completely at week 4 (Figure 1(c)). Flow cytometric analysis demonstrated that <1% early EPCs expressed CD11b, a monocyte/macrophage marker (data not shown). Uptake of Dil-Ac-LDL and binding of FITC-UEA-lectin showed that over 80% early EPCs were Dil-Ac-LDL/FITC-UEA-lectin double positive (Figure 1(d), cells uptaking Dil-Ac-LDL showed red.  Figure 1(e), cells positive for FITC-UEA-lectin appeared green. Figures 1(f), 1(d), and 1(e) merged, orange was double positive). Western blotting analysis showed that late EPCs produced a group of endothelial protein markers, that is, KDR/VEGFR2, Tie2, and eNOS (Figures 1(g), 1(h), and 1(i), resp.) with the Tie2 level relatively lower in EPCs than that in HUVECs while the other 2 protein levels comparable to those detected in HUVECs.

### 3.2. Upregulation of IL-1*β* Expression in THP-1 Monocytic Cells Cocultured with Late Human EPCs

**Figure 2:**
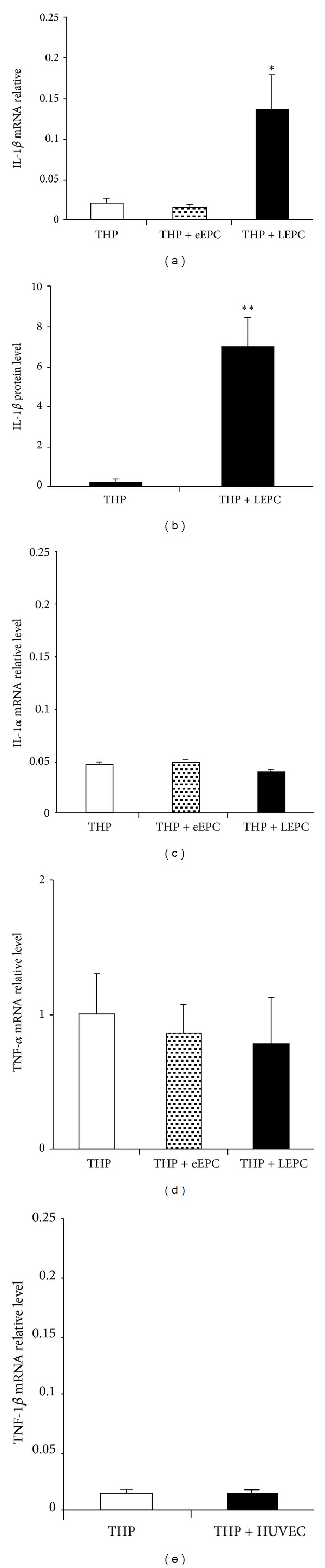
Late human EPCs upregulated IL-1*β* expression in THP-1 monocytic cells. Coculture with late but not early EPCs led to a significant increase of IL-1*β* mRNA levels in THP-1 cells as assayed by real-time RT-PCR with GAPDH as internal control (a). The conditioned medium (CM) from the late EPCs/THP-1 cell coculture system contained a significant higher level of IL-1*β* protein as measured by a high sensitivity ELISA Kit (b). In contrast, neither early nor late EPCs affected IL-1*α* (c) or TNF-*α* expression (d). Coculture with terminally differentiated HUVECs did not result in changes of IL-1*β* mRNA levels in THP-1 cells (e). *P < 0.01 (*n* = 5), **P < 0.005 (*n* = 5), both compared to THP-1 cells cultured alone. eEPC: early EPC; LEPC: late EPC.

To examine if EPCs regulate IL-1*α*, IL-1*β*, and TNF-*α* gene expression in monocytes, human THP-1 monocytic cells were cocultured with early or late human EPCs. mRNA levels of IL-1*α*, IL-1*β*, and TNF-*α* in THP-1 cells were assayed by real-time RT-PCR, and IL-1*β*, protein in coculture conditioned medium was further measured by a high sensitivity ELISA Kit (R&D Systems). The results showed no significant difference in IL-1*β* mRNA levels between THP cells cultured alone and THP-1 cells cocultured with early EPCs (Figure 2(a)). However, coculture with late EPCs led to a significant increase of IL-1*β* mRNA levels (0.134 ± 0.046), compared to THP-1 cells cultured alone (0.019 ± 0.007, *n* = 5, *P* < 0.01, Figure 2(a)). Furthermore, the increased IL-1*β* mRNA levels were reflected by the elevated IL-1*β*, protein levels in the coculture conditioned medium as determined by an ELISA Kit (0.257 ± 0.223 pg/mL for THP-1 cell culture alone versus 6.933 ± 1.501 pg/mL for THP-1 cells cocultured with late EPCs, Figure 2(b), *n* = 5, *P* < 0.005). No significant changes were observed for IL-1*α* or TNF-*α* mRNA levels following THP-1 cell coculture with either early or late EPCs (Figures 2(c) and 2(d)). We further examined if terminally differentiated HUVECs had similar effect as late EPCs and found that coculture with HUVECs did not result in significant alternation in IL-1*β* mRNA levels in THP-1 cells (Figure 2(e)).

### 3.3. Upregulation of IL-1*β* Expression in THP-1 Cells Is Afforded by Late EPCs Partly through a Paracrine Manner

**Figure 3:**
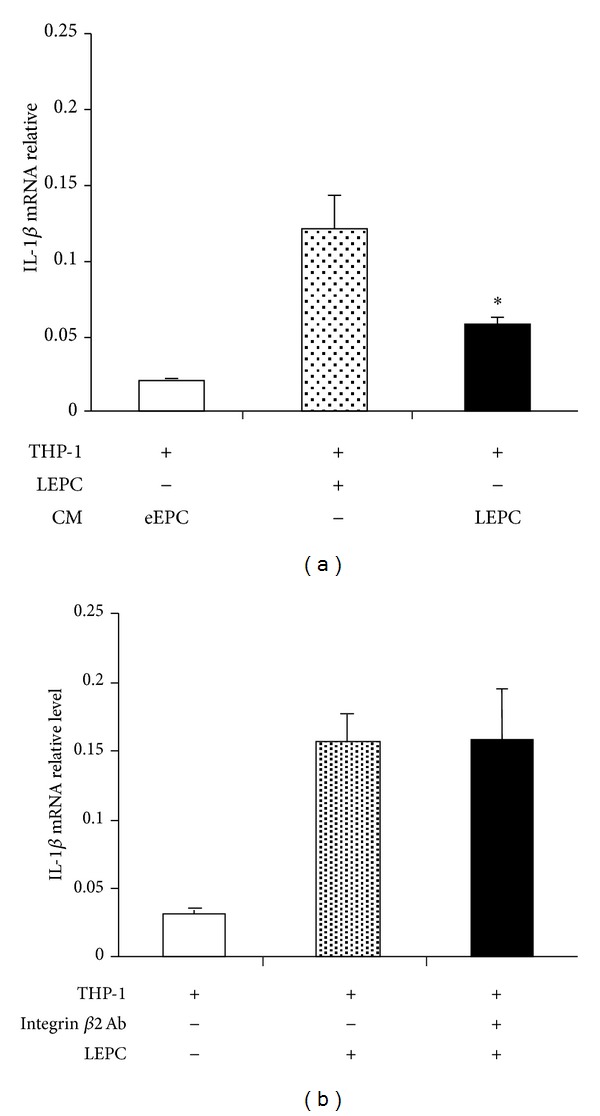
Late human EPCs augmented IL-1*β* expression through a paracrine pathway. THP-1 cells were cultured in the presence of late EPC conditioned medium (CM) with early EPC CM serving as control, and then IL-1*β* mRNA levels in THP-1 cells were determined by real-time RT-PCR. The results showed that late EPC CM was able to partly elevate IL-1*β* mRNA levels (a). THP-1 cells were preincubated with a blocking antibody against integrin *β*2 followed by coculture with late EPCs, which however did not abolish the effect of late EPC stimulated IL-1*β* mRNA increase (b). *P < 0.05 (*n* = 5), compared to THP-1 cells treated with early EPC CM. eEPC: early EPC; LEPC: late EPC.

The mechanisms of late EPCs upregulating IL-1*β* expression were further explored. Addition of late EPC CM to THP-1 cell culture (with early EPC CM as control) resulted in a moderate increase in IL-1*β* mRNA levels (0.057 ± 0.014), lower than that of THP-1 cell/late EPC coculture (0.120 ± 0.050) but significantly higher than 0.020 ± 0.004 of IL-1*β* mRNA levels in THP-1 cells treated with early EPC CM (*n* = 5, *P* < 0.05, Figure 3(a)), suggesting a paracrine effect afforded by late EPCs. It has been shown that activation of integrin *β*2 on neutrophils augments IL-1*β* expression. As THP-1 cells produce integrin *β*2, we examined whether integrin *β*2 is involved in late EPC regulated IL-1*β* expression. Preincubation of THP-1 cells with an integrin *β*2 blocking antibody did not abolish late EPC effect on IL-1*β* mRNA production (Figure 3(b)).

## 4. Discussion

In the present study, we examined the effect of EPCs on IL-1*α*, IL-1*β* and TNF-*α* expression in monocytes using coculture of human EPCs and THP-1 monocytic cells. Human early and late outgrowth EPCs were procured by culture of PBMNCs in endothelial selective medium EGM-2. Uptake of Dil-Ac-LDL and binding of FITC-UEA-lectin demonstrated over 80% early EPCs were Dil-Ac-LDL/FITC-UEA-lectin double positive, which is in agreement with previously published data [11, 21]. Late EPCs expressed important endothelial protein markers, that is, KDR/VEGFR2, Tie2, and eNOS, as detected by western blotting. When THP-1 monocytic cells were cocultured with early EPCs, none of the 3 cytokines expression in THP-1 cells was altered. In contrast, late EPCs significantly upregulated IL-1*β* expression at both mRNA and protein levels but did not affect IL-1*α* or TNF-*α* expression. ELISA results for IL-1*α* or TNF-*α* protein assay (data not shown) were in agreement with the mRNA data. As IL-1*β* mRNA was barely measured by real-time RT-PCR in late EPCs (data not shown), the possibility of contaminating IL-1*β* mRNA from late EPCs was excluded in the coculture system. As late EPCs demonstrate similar morphology, surface protein marker expression, and proliferative ability as somatic ECs, we examined whether ECs have an effect on IL-1*β* expression. Coculture with HUVECs, however, did not lead to changes in IL-1*β* expression in THP-1 cells. In this regard, late EPCs differ from HUVECs.

The mechanisms by which EPCs upregulate IL-1*β* expression in THP-1 cells were further investigated. Walzog et al. observed that activation of integrin *β*2 in human neutrophils resulted in augmentation of IL-1*β* synthesis [22]. It has been well documented that THP-1 cells express integrin *β*2 [23, 24]. We explored whether integrin *β*2 was involved in the regulation of IL-1*β* expression in THP-1 cells. Pre-incubation of THP-1 cells with an integrin *β*2 blocking antibody did not abolish the effect of late EPCs on IL-1*β* mRNA production, suggesting that regulation of IL-1*β* expression in THP-1 cells by late EPCs is independent of integrin *β*2. When late EPC CM was added to THP-1 cell culture, a modest 3-fold increase of IL-1*β* mRNA levels was detected compared to early EPC CM-treated or nontreated cells, indicating that late EPCs up-regulate IL-1*β* production, in part, through a paracrine mechanism.

While considered a critical inflammatory mediator [25], IL-1*β* has also been shown to play an important role in angiogenesis and modulation of EPC function [26–29]. Using the matrigel plug assay, Voronov et al. observed that vascularization of the plugs was present in wild type mice, but was absent in IL-1*β* knockout mice [26], suggesting a role for IL-1*β* in angiogenesis. In vitro studies have shown that treatment of murine EPCs with IL-1*β* increases EPC numbers, and IL-1*β* treated EPCs form a significantly higher number of vessel-like structures compared to nontreated cells after culture in matrigel-coated dishes [27]. Yang et al. have observed that, in addition to stimulating EPC proliferation, IL-1*β* promotes EPC migration and adhesion and upregulates the production of VEGF-A in EPCs [28]. Using a murine model of ischemic hind limb, Amano et al. reported that the number of circulating EPCs in IL-1*β*−/− mice was significantly lower than in wild type littermates following hind limb ischemia. EPC numbers in the ischemic muscle were also significantly reduced in IL-1*β*−/− mice [29], indicating a crucial role for IL-1*β* in ischemia-induced EPC mobilization and homing. Increased IL-1*β* production in monocytes induced by late EPCs, as we reported in this study, could therefore form a self-amplifying loop for EPCs, which might represent a yet unidentified endogenous mechanism that improves EPC recruitment and function required by tissue repair and regeneration.

## 5. Conclusion

In conclusion, in a human EPC/THP-1 monocytic cell coculture system, late but not early EPCs upregulate IL-1*β* expression in THP-1 cells, which is partly through a paracrine pathway. IL-1*β*, an important inflammation mediator, has also been shown to promote EPCs proliferation, migration, and adhesion. Our data therefore suggest that EPCs can exert self-enhancement effects by interacting with monocytes and that EPCs might also modulate inflammatory reactions by regulating IL-1*β* expression in monocytes.
